# Potato steroidal glycoalkaloids: properties, biosynthesis, regulation and genetic manipulation

**DOI:** 10.1186/s43897-024-00118-y

**Published:** 2024-12-13

**Authors:** Yongming Liu, Xiaowei Liu, Yingge Li, Yanfei Pei, Abdul Jaleel, Maozhi Ren

**Affiliations:** 1 Institute of Urban Agriculture, Chinese Academy of Agricultural Sciences, Chengdu National Agricultural Science and Technology Center, Chengdu, 610213 China; 2 Yazhouwan National Laboratory, Sanya, 572025 China; 3 Chengdu Agricultural College, Chengdu, 611130 China; 4 Hainan Seed Industry Laboratory, Sanya, 572025 China; 5 Department of Integrative Agriculture, College of Agriculture and Veterinary Medicine, United Arab Emirates University, P.O. Box 15551, Al Ain, United Arab Emirates

**Keywords:** Potato, Steroidal glycoalkaloids, Antinutritional factors, Biosynthesis, Regulation network, Quality improvement, Toxicity

## Abstract

Steroidal glycoalkaloids (SGAs), predominantly comprising α-solanine (C_45_H_73_NO_15_) and α-chaconine (C_45_H_73_NO_14_), function as natural phytotoxins within potatoes. In addition to their other roles, these SGAs are crucial for enabling potato plants to withstand biotic stresses. However, they also exhibit toxicity towards humans and animals. Consequently, the content and distribution of SGAs are crucial traits for the genetic improvement of potatoes. This review focuses on advancing research related to the biochemical properties, biosynthesis, regulatory mechanisms, and genetic improvement of potato SGAs. Furthermore, we provide perspectives on future research directions to further enhance our understanding of SGA biosynthesis and regulation, ultimately facilitating the targeted development of superior potato varieties.

## Introduction

Potato (*Solanum tuberosum*) ranks as the world’s third-largest food crop, surpassed only by rice and wheat. It is one of the most widely cultivated foods, grown in over 140 countries. Renowned for its high yields, resilience to barren conditions, robust resistance to adverse environments, substantial economic benefits, and an extensive industrial chain, potato plays a pivotal role in ensuring global food security (Devaux et al. 2020; Qu et al. 2024).

SGAs are specialized metabolites produced by numerous *Solanum* species, including popular vegetable crops such as tomatoes, potatoes, and eggplants. Extensive research has established that SGAs play crucial roles in plant defense, and many of these compounds exhibit documented anti-cancer, anti-microbial, anti-inflammatory, anti-viral, and anti-pyretic activities (Lucier et al. 2024). However, certain SGAs have anti-nutritional effects on humans, significantly impacting the post-harvest quality and food safety of potato. Notably, SGAs are known to inhibit acetylcholinesterase activity, adversely affecting the nervous and digestive systems of vertebrates (Dhalsamant et al. 2022). Consuming potato tubers with SGAs levels exceeding 3 mg/kg body weight can induce symptoms such as nausea, vomiting, and diarrhea in humans, and in severe cases, may lead to death owing to gastrointestinal and neurological disorders (Friedman. 2006; Zhao et al. 2021). The generally acceptable limit for total glycoalkaloid (TGA) content in tubers for human consumption is 200 mg/kg of fresh weight (FW).

SGAs currently present two main challenges to the advancement of the potato industry. First, they impact the edible quality and nutritional value of potato tubers. Through extensive selective breeding and domestication, the SGA content in modern cultivars has been reduced to a safe level, generally posing minimal health risks upon consumption. However, exposure to light, mechanical damage, sprouting, insect infestation, or localized decay during post-harvest processes such as transportation, storage, and marketing can trigger the significant synthesis and accumulation of SGAs (Benkeblia, 2020; Shen et al. 2022; Hu et al. 2023). Due to the high melting point of SGAs, which is up to 284 °C, conventional cooking methods like steaming, baking, deep-frying, and brief microwave treatment are insufficient for effective degradation. Reports of food poisoning caused by the consumption of potatoes with excessive SGA levels are fairly common. Furthermore, livestock can still suffer poisoning from consuming steamed sprouted potatoes (Jayanty et al. 2019). The safety hazards of SGAs to humans and animals pose a great challenge to potato production for farmers, impacting their annual income (Tajner-Czopek et al. 2015). In countries and regions lacking refrigeration and cold-chain logistics, quality deterioration of potatoes due to SGA accumulation can affect 30–40% of the total yield (Nie et al. 2019).

Secondly, SGAs restrict the diversity of materials used for potato cultivation. The potato varieties ‘Lenape’ and ‘Magnum Bonum’, once widely cultivated in the United States and Sweden, respectively, had to be withdrawn from the market due to high levels of SGAs in their tubers. Among 99 newly introduced or developed potato lines in Yunnan province of China, more than 12% exceeded the safe consumption range for solanine levels (Huang et al. 2011). Measurement of SGA content (the sum of α-solanine and α-chaconine) in 10 diploid wild potato species revealed that all their flesh exceeded the safety limit, with the lowest reaching 300 mg/kg FW and the highest surpassing 1600 mg/kg FW (Peng et al. 2019). Due to the high level of SGAs, these new lines and wild potato materials face challenges in being directly adopted for agricultural production. In summary, the high SGA content in tubers not only poses a significant threat to the edible quality and food safety of potatoes but also hinders the industrial development of the potato sector. Therefore, exploring the biochemical properties and metabolic patterns of SGAs in potatoes is essential for ensuring food safety and maintaining postharvest quality.

## Biochemical properties of potato SGAs

### Chemical structures of SGAs

Currently, SGAs have been identified in various species, with more than 80 types discovered in potatoes. Among these, α-solanine (C_45_H_73_NO_15_) and α-chaconine (C_45_H_73_NO_14_) are the primary components, with a ratio ranging from 1:2 to 1:7, making up more than 90% of the total glycoside alkaloids (Sonawane et al. 2020) (Table 1). Additionally, a survey of 12 cultivated potato varieties revealed that minor SGAs, including solasonine, solamargine, iminiumsolanine, and iminiumchaconine, are all present at levels below 0.9 mg/kg FW in tubers (Baur et al. 2022). The chemical structures of α-solanine and α-chaconine are highly similar, sharing the same glycoside aglycone, solanidine, but differing in their glycosidic units. The characteristic structures of SGAs are defined by their types of steroidal aglycones and glycoside residues (Heinig et al. 2014). Steroidal glycosides can generally be classified into two types: spirosolane and solanidane, with spirosolane being the most common form in *Solanaceae* plants (Milner et al. 2011). SGAs share a common steroidal skeleton, and the presence (unsaturated) or absence (saturated) of a double bond at the C-5,6 position in the steroidal alkaloid glycosides contributes to their structural diversity. Solanidine-type aglycones, such as solanidine, are predominantly found in cultivated and wild potato species. The most common glycoside molecules that modify the structure of SGAs include lycotetraose, composed of a single *D*-xylose, *D*-galactose, and two *D*-glucoses; solatriose, composed of *D*-galactose, *D*-glucose, and *L*-rhamnose; chacotriose, composed of a single *D*-galactose and two *L*-rhamnoses; and commertetraose, composed of three *D*-glucoses and one *D*-galactose (Table 1) (Heretsch et al. 2015; Sonawane et al. 2020; Zhao et al. 2021).

**Table 1:** Several common glycoalkaloids identified in potatoes

Name	CAS accession number	PubChem CID	Glycosidic unit	Molecular formula	Chemical structure components	First reported species donor
α-Chaconine	20562-03-2	442,971	β-Chacotriose	C_45_H_73_NO_14_	Solanidine + glucose + rhamnose + rhamnose	*S. chacoense*
α-Solamarine	20318-30-3	70,680,623	β-Solatriose	C_45_H_73_NO_16_	Tomatidenol + galactose + glucose + rhamnose	*S. tuberosum* L. var Kennebec
α-Solanine	20562-02-1	9,549,171	β-Solatriose	C_45_H_73_NO_15_	Solanidine + galactose + glucose + rhamnose	*S. nigrum*
α-Tomatine	17406-45-0	5513	β-Lycotetrose	C_50_H_83_NO_21_	Tomatidine + glucose + glucose + galactose + xylose	*S. lycopersicum*
β-Chaconine	472-51-5	119,393	β-Chacobiose	C_39_H_63_NO_10_	Solanidine + glucose + rhamnose	
β-Solamarine	3671-38-3	168,971	β-Chacotriose	C_45_H_73_NO_15_	Tomatidenol + glucose + rhamnose + rhamnose	*S. tuberosum* L. var Kennebec
β-Solanine	61877-94-9	45,479,590	β-Solabiose	C_39_H_63_NO_11_	Solanidine + galactose + glucose	
γ-Chaconine	511-36-4	21,123,844	Glucose	C_33_H_53_NO_6_	Solanidine + glucose	
γ-Solanine	511-37-5	20,841,681	Galactose	C_33_H_53_NO_6_	Solanidine + galactose	
Commersonine	60776-42-3	185,997	β-Commertetraose	C_51_H_85_NO_21_	Demissidine + galactose + glucose + glucose + glucose	*S. commersonii*
Dehydrocommersonine	65428-74-2	101,699,426	Β-Commertetraose	C_51_H_83_NO_21_	Solanidine + galactose + glucose + glucose + glucose	*S. commersonii*
Dehydrodemissine	195433-57-9	131,751,363	β-Lycotetraose	C_50_H_81_NO_20_	Solanidine + galactose + glucose + glucose + xylose	*S. commersonii*
Demissine	6077-69-6	442,975	β-Lycotetraose	C_50_H_83_NO_20_	Demissidine + galactose + glucose + glucose + xylose	*S. chacoense*
Leptine I	101030-83-5	180,940	β-Chacotriose	C_47_H_75_NO_16_	O(23)-Acetylleptinidine + glucose + rhamnose + rhamnose	*S. chacoense*
Leptine II	101054-39-1	101,699,888	β-Solatriose	C_47_H_75_NO_17_	O(23)-Acetylleptinidine + galactose + glucose + rhamnose	*S. chacoense*
Leptinine I	101009-59-0	101,699,423	β-Chacotriose	C_45_H_73_NO_15_	Leptinidine + glucose + rhamnose + rhamnose	*S. chacoense*
Leptinine II	100994-57-8	101,699,425	β-Solatriose	C_45_H_73_NO_16_	Leptinidine + galactose + glucose + rhamnose	*S. chacoense*
Solamargine	20311-51-7	73,611	β-Chacotriose	C_45_H_73_NO_15_	Solasodine + glucose + rhamnose + rhamnose	*S. berthaultii*
Solasonine	19121-58-5	119,247	β-Solatriose	C_45_H_73_NO_16_	Solasodine + galactose + mannose + glucose	*S. berthaultii*

### Biological functions of potato SGAs

**Fig. 1:**
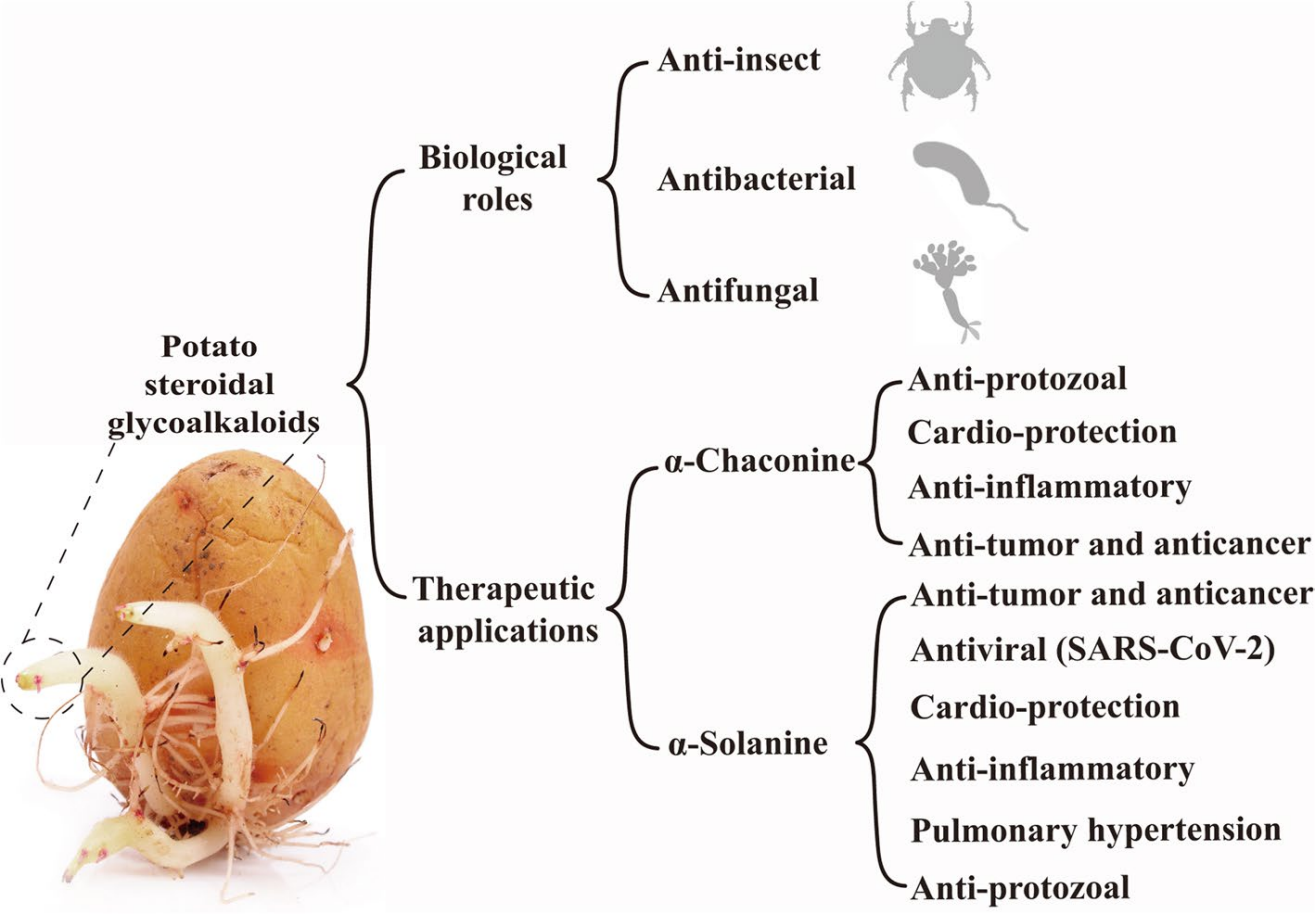
Biological roles and therapeutic applications of potato steroidal glycoalkaloids (SGAs). The therapeutic applications of α-solanine and α-chaconine were referenced from (Delbrouck et al. 2023). The image of the potato tuber is credited to Shanghai Hanzhong Network Technology Co., Ltd

SGAs in potatoes possess antibacterial, antifungal, and insecticidal properties, providing protective effects for the plant and holding significant potential for application in potato breeding to enhance resistance to diseases and pests (Fig. 1). Potato SGAs exhibit significant and board inhibitory activity against various fungi. Globally, potato dry rot caused by more than 13 species of *Fusarium* poses a major threat during potato storage. In vivo and in vitro experiments have demonstrated that crude extracts of SGAs isolated from potato tuber peels can significantly inhibit the growth of *Fusarium sulphureum*, with the growth inhibition rate positively correlated with the concentration of SGAs (Li et al. 2023). Further studies have revealed that the inhibitory activity of potato SGAs against *Fusarium solani* is primarily achieved by altering energy metabolism pathways, such as the tricarboxylic acid cycle, hexokinase activity, ATPase activity, and mitochondrial complex activity in the fungus (Zhang et al. 2023, 2024). In vitro inhibition tests showed that, when mixed at a total concentration of 500 µM and a ratio of 3:1 of α-chaconine to α-solanine, the inhibition rates for *F. verticillioides* and *F. graminearum* reached 15–20%, demonstrating an effect comparable to the commonly used antifungal organic solvent, 8% N, N-dimethylformamide (DMF) (Pacifico et al. 2024). Late blight caused by *Phytophthora infestans* is one of the major diseases affecting potatoes and was responsible for the Irish Potato Famine. The content of SGAs in tubers is significantly upregulated after infection by *Phytophthora infestans*. Zoospore-mobility test experiments found that four SGAs, including α-solanine, α-chaconine, solasonine, and solamargine, exhibited notable inhibitory effects on the motility of *Phytophthora infestans* zoospores in vitro, with IC50 values ranging from 10 to 47 µM (Baurnicole et al. 2022). However, some studies have indicated that α-solanine and α-chaconine do not have significant direct inhibitory effects on the mycelial growth of *Phytophthora infestans*, whereas their non-glycosylated precursor, solanidine, exhibits strong inhibitory effects (Dahlin et al. 2017). Although α-solanine and α-chaconine have similar structures, α-chaconine demonstrates stronger and broader-spectrum antifungal activity. It can effectively inhibit the growth of strains such as *Mucor plumbeus* FUA5003, *Mycosphaerella pinodes* Is.39, *Alternaria alternata* AA001, *Pyrenophora teres* f. *teres* SK51, and *Pyrenophora tritici*-*repentis* 331-2 by directly interacting with membrane sterols through its sugar moiety to disrupt the fungal membrane (Maldonado et al. 2016). In addition to fungi, potato SGAs also exhibit inhibitory effects on pathogens such as *Staphylococcus aureus*, *Pseudomonas aeruginosa*, and *E.* coli (Ismail et al. 2022).

α-Solanine and α-chaconine exhibit toxicity to various pests, including *Galleria mallonella* (Büyükgüzel et al. 2013), *Macrosiphum euphorbiae* (Güntner et al. 2000), *Myzus persicae* (Fragoyiannis et al. 1998), *Tribolium castaneum* (Nenaah. 2011), *Trogoderma granarium* (Nenaah. 2011), and *Zophobas atratus* (Ventrella et al. 2015). These compounds act as cell membrane disruptors or inhibitors of acetylcholinesterase activity, leading to reduced fecundity and feeding, weight loss, and increased mortality in the affected pests. Additionally, some unique SGAs found in wild potatoes, such as leptinines, commersonine, dehydrocommersonine, demissine, and dehydrodemissine, confer natural resistance to pathogens and the Colorado potato beetle (Tai et al. 2014; Cárdenas et al. 2019; Wolters et al. 2023).

Researchers have been investigating the potential medicinal value of potato SGAs due to their unique biological activities. Recent discoveries have revealed that potato glycoalkaloids exhibit a range of therapeutic properties, including anti-tumor, antiviral, and antioxidant effects (Fig. 1). A previous review (Delbrouck et al. 2023) comprehensively summarized these medicinal values. The characteristics and applications of these compounds in anti-tumor and anticancer contexts have attracted considerable interest. Another recent review (Manoharan et al. 2024) extensively discusses the functional mechanisms of potato SGAs in these contexts.

## SGAs biosynthesis in potato

### Effects of genetic and environmental factors

**Fig. 2:**
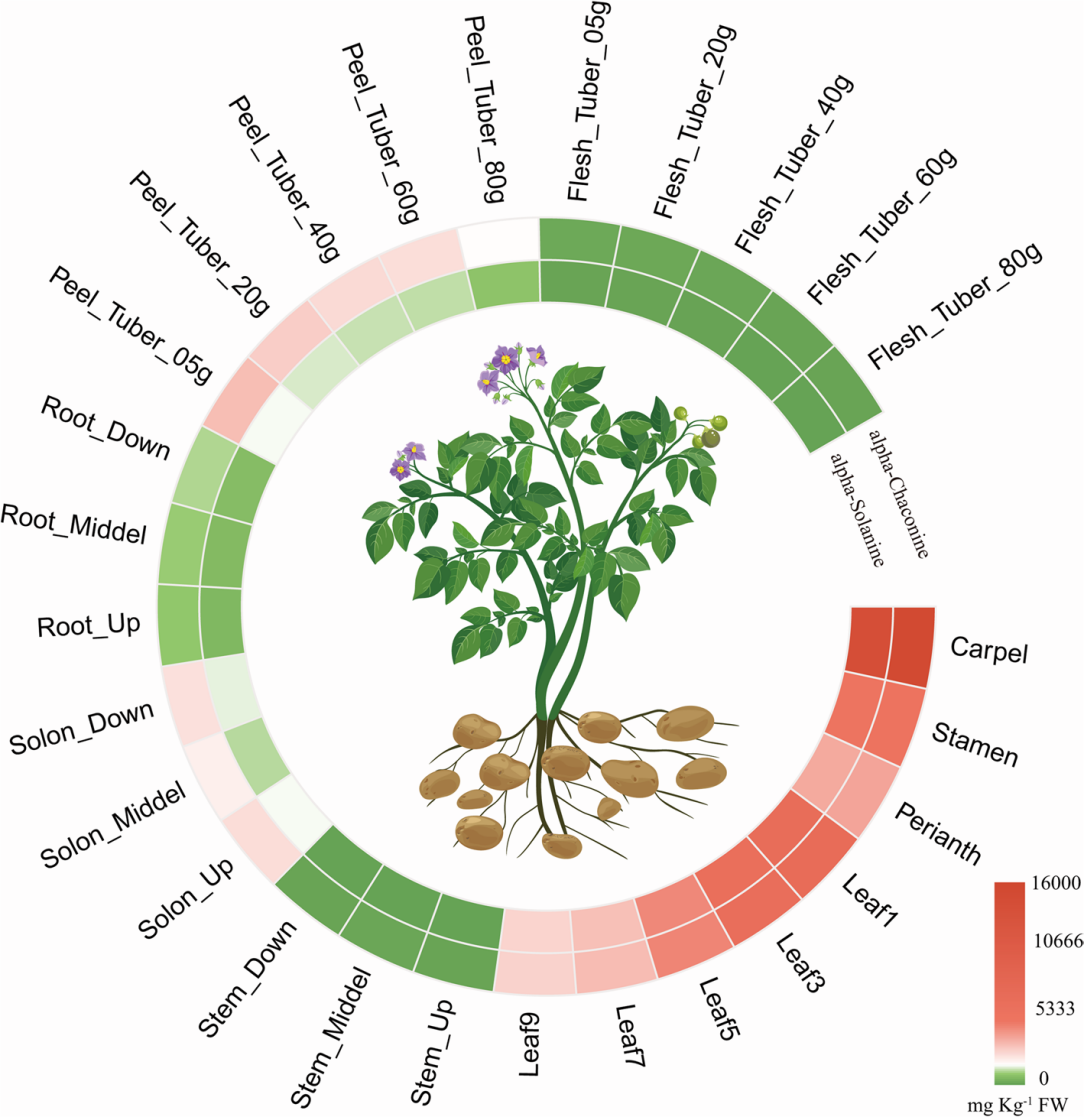
Accumulations of α-solanine and α-chaconine in different organs or tissues of the diploid clone RH. Leaf1 to Leaf9 represent samples from the first to the ninth expanded leaves. Data were retrieved from (Peng et al. 2019). The image of the potato plant is credited to Shanghai Hanzhong Network Technology Co., Ltd

The levels of SGAs in potatoes are influenced by genetic and environmental factors, including genotype, tissue type, and environmental conditions. SGAs exhibit high heritability, ranging from 86 to 89% (broad sense) and 66–84% (narrow sense) (Benkeblia, 2020). A comparison of SGA content in the tubers of 10 diploid wild species, 10 diploid landraces, and 2 tetraploid modern cultivated varieties revealed no significant difference in the SGA content of tuber peel between wild and cultivated species. However, the SGA content in the tuber flesh of wild potato was significantly higher than that of cultivated potato, far exceeding the safety threshold of 200 mg/kg FW (Peng et al. 2019). This suggests that during the domestication of potatoes, SGAs in edible organs were removed or reduced, with the genes regulating SGA content being key genes for domestication (Hardigan et al. 2017). The SGA content varies greatly among different tissues, with α-solanine and α-chaconine being more concentrated in organs or tissues such as flowers, leaves, tuber peel, stolons, and roots, while their levels are relatively low in flesh and stems (Fig. 2) (Peng et al. 2019). Many environmental factors, such as mechanical damage, adverse storage conditions (low temperature and strong light), and processing conditions can significantly increase the SGA content in potatoes (Haase. 2010; Petersson et al. 2013; Nie et al. 2018; Baur et al. 2022; Merino et al. 2023). Different varieties show distinct responses to these conditions, indicating the presence of genotype-environment interactions in SGA synthesis.

### Biosynthesis routines of SGAs

The synthesis pathway of SGAs is divided into two segments: the pre-cholesterol pathway and the post-cholesterol pathway, with cholesterol serving as an intermediate marker. The pre-cholesterol pathway is a common component in the sterol synthesis of all plants, while the post-cholesterol pathway is essential for generating diverse SGAs both within and among plant species. In the pre-cholesterol pathway, acetyl-coenzyme A (acetyl-CoA) serves as the initial substrate, leading to the formation of isoprenoid pyrophosphate (IPP) and dimethylallyl diphosphate (DMAPP) via the mevalonate (MVA) pathway. Subsequently, enzymes including squalene synthase (SQS), squalene epoxidase (SQE), and cycloartenol synthase (CAS) facilitate the formation of cycloartenol. In plants, cycloartenol functions as a branching point for two parallel metabolic pathways: one leading to the C-24 alkyl phytosterols pathway, and the other, catalyzed by sterol side chain reductase 2 (SSR2, also known as DWF1-L), leading to the cholesterol synthesis pathway (Sawai et al. 2014). Identified duplicate genes between the two pathways include Δ24-sterol reductase-like (*DWF1*-L) and *DWF1*, sterol C4-methyl oxidase 1-like (*SMO1*-L) and *SMO1*, ∆7-sterol C5-desaturase-like (*DWF7*-L) and *DWF7*, ∆7-sterol reductase-like (*DWF5*-L) and *DWF5* (Sawai et al. 2014; Sonawane et al. 2016; Nahar et al. 2017).

**Fig. 3:**
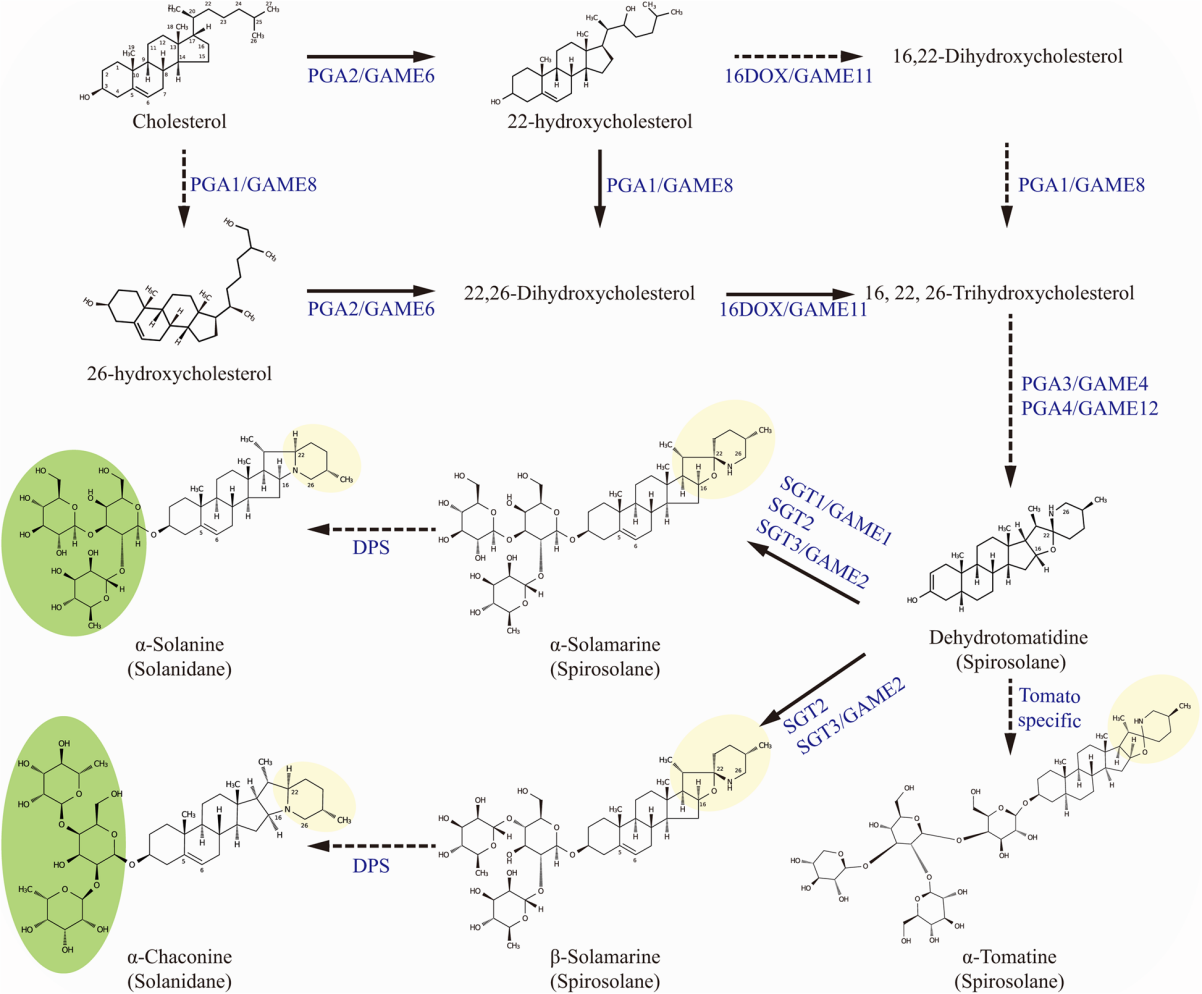
Proposed biosynthetic pathway of steroidal glycoalkaloids (SGAs) in potatoes. The figure illustrates the key steps involved in SGA biosynthesis, with solid arrows representing confirmed reaction stages and dashed arrows indicating steps that require further elucidation. The diagram highlights the distinct glycosidic units between α-solanine and α-chaconine, denoted by a green background, and emphasizes the crucial chemical differences between spiro- and solanine-type SGAs, indicated by a yellow background. For a comprehensive understanding of each step, please refer to the detailed description in the main text

The post-cholesterol pathway involves the conversion of cholesterol into various SGAs (Fig. 3). Cholesterol undergoes a series of hydroxylations catalyzed by cytochrome P450 monooxygenases (CYPs) to form 16, 22, 26-trihydroxycholesterol. Specifically, Glycoalkaloid Metabolism 6 (GAME6)/PGA2, GAME8/PGA1, and GAME11/16DOX are responsible for hydroxylation at the C-22, C-26, and C-16 positions, respectively. Following oxidation by GAME4/PGA3, a nitrogen atom is introduced at the C-26 position by the transaminase GAME12/PGA4, leading to the formation of dehydrotomatidine (Itkin et al. 2013; Umemoto et al. 2016; Nakayasu et al. 2017; Grzech et al. 2024). Subsequently, various glycosidic units are added to the aglycone through the action of UDP-glycosyltransferases (UGTs) such as solanidine galactosyltransferase 1 (SGT1), SGT2, and SGT3, resulting in the structural differences between α-solanine and α-chaconine (Mccue et al. 2007, 2018). After glycosylation, Dioxygenase for Potato Solanidane synthesis (DPS) catalyzes the ring arrangement from spirosolanes to solanidanes (Akiyama et al. 2021).

For certain wild potatoes, such as *Solanum chacoense*, α-solanine and α-chaconine can be hydroxylated by their unique 2-oxoglutarate-dependent dioxygenases (ScGAME32) to form leptinines (leptinine I and leptinine II), and further acetylated to ultimately produce leptines (leptine I and leptine II). These modified SGAs exhibit insecticidal activity against the Colorado potato beetle (Cárdenas et al. 2019). Currently, most of the catalytic enzyme genes in the SGA biosynthetic pathway have been cloned and identified. These primarily include 3-hydroxy-3-methylglutaryl-coenzyme A reductase (HMGR1), squalene synthase (PSS1, SQE), C5-desaturase (C5-SD/DWF7-L), 16α-hydroxylase (16DOX), various hydroxylases (GAME4, GAME6, GAME8, GAME11), transaminase (GAME12), and glycosyltransferases (SGT1, SGT2, SGT3). Like other plant metabolic gene clusters (Cao et al. 2024), it was discovered that *SGT3*, *GAME11*, *GAME6*, and *SGT1* are clustered and tandemly arranged on chromosome 7, while *GAME12* and *GAME4* are adjacent and arranged on chromosome 12 (Table 2) (Itkin et al. 2013; Sonawane et al. 2016). Furthermore, it is noteworthy that the *DPS* branch genes are highly duplicated in tandem on potato chromosome 1 (Cárdenas et al. 2019).

**Table 2:** Genetic factors involved in potato steroidal glycoalkaloids (SGAs) metabolism

Gene name	Locus	Family	Chromosome	Gene start (bp)	Gene end (bp)	Strand	References
*GAME33-1*	Soltu.DM.01G002170	Dioxygenase	chr01	2,290,038	2,292,302	+	(Sonawane et al. 2022)
*GAME33*	Soltu.DM.01G002270	Dioxygenase	chr01	2,453,150	2,456,358	+	(Sonawane et al. 2022)
*DPS*	Soltu.DM.01G002240	Dioxygenase	chr01	2,411,119	2,413,818	-	(Akiyama et al. 2021)
*JRE4/GAME9*	Soltu.DM.01G031000	AP2/ERF transcription factor	chr01	70,710,298	70,711,462	-	(Cárdenas et al. 2016; Thagun et al. 2016)
*SMO1-L*	Soltu.DM.01G031960	Sterol C4-methyl oxidase 1-like	chr01	71,798,695	71,801,666	-	(Merino et al. 2023)
*HMGR1*	Soltu.DM.02G004910	3-Hydroxy-3-methylglutaryl-coenzyme A reductase	chr02	17,738,149	17,740,939	-	(Suzuki et al. 2004)
*SSR2/DWF1-L*	Soltu.DM.02G012480	Cycloartenol reductase	chr02	27,221,284	27,231,507	-	(Sawai et al. 2014; Nahar et al. 2017)
*C5-SD/DWF7-L*	Soltu.DM.02G026060	Sterol C-5(6) desaturase	chr02	39,146,404	39,149,183	+	(Merino et al. 2023; Li et al. 2024)
*CAS*	Soltu.DM.04G019820	Cycloartenol synthase	chr04	45,889,860	45,900,069	-	(Corey et al. 1993)
*SQE*	Soltu.DM.04G032150	Squalene epoxidase	chr04	63,771,283	63,775,215	-	(Manrique-Carpintero et al. 2013)
*SMO2-L*	Soltu.DM.06G003760	Sterol C-4 methyl oxidase 2-like	chr06	4,549,750	4,554,522	+	(Merino et al. 2023)
*PGA1/GAME8*	Soltu.DM.06G018370	Hydroxylase	chr06	44,819,947	44,822,047	-	(Umemoto et al. 2016)
*DWF5-L*	Soltu.DM.06G029480	∆7-Sterol reductase-like	chr06	54,639,699	54,649,830	*+*	(Merino et al. 2023)
*SGT3/GAME2*	Soltu.DM.07G014160	β-Solanine/β-Chaconine rhamnosyl transferase	chr07	43,432,520	43,434,341	-	(Cárdenas et al. 2016; Tsukagoshi et al. 2016)
*16DOX/GAME11*	Soltu.DM.07G014170	2-Oxoglutarate dioxygenase	chr07	43,555,537	43,557,153	+	(Cárdenas et al. 2016; Tsukagoshi et al. 2016)
*PGA2/GAME6*	Soltu.DM.07G014190	Hydroxylase	chr07	43,594,461	43,596,848	+	(Cárdenas et al. 2016; Tsukagoshi et al. 2016)
*SGT1/GAME1*	Soltu.DM.07G014220	Solanidine galactosyltransferase	chr07	43,662,699	43,664,480	+	(Mccue et al. 2006, 2018; Cárdenas et al. 2016)
*SGT2*	Soltu.DM.08G022920	Solanidine glucosyltransferase	chr08	52,338,169	52,340,193	-	(Mccue et al. 2006, 2018; Manrique-Carpintero et al. 2013)
*PSS1/SQS1*	Soltu.DM.10G016360	Squalene synthase	chr10	45,903,711	45,909,486	+	(Krits et al. 2007; Ginzberg et al. 2012)
*StMYB113*	Soltu.DM.10G020780	MYB transcription factor	chr10	52,388,848	52,391,237	**-**	(Liu et al. 2024)
*PGA3/GAME4*	Soltu.DM.12G024040	Hydroxylase	chr12	53,896,395	53,904,071	-	(Cárdenas et al. 2016; Tsukagoshi et al. 2016)
*PGA4/GAME12*	Soltu.DM.12G024050	γ-Amino butyric acid transaminase	chr12	53,969,804	53,977,550	+	(Cárdenas et al. 2016; Tsukagoshi et al. 2016; Nakayasu et al. 2021)
*ScGTR1*,* ScGTR2*	/	Glycosyltransferases	/	/	/	/	(Wolters et al. 2023)
*ScGAME32*	/	Dioxygenases	/	/	/	/	(Cárdenas et al. 2019)

## Regulatory network of steroidal glycoalkaloids synthesis in potato

### Transcription factors (TFs)

**Fig. 4:**
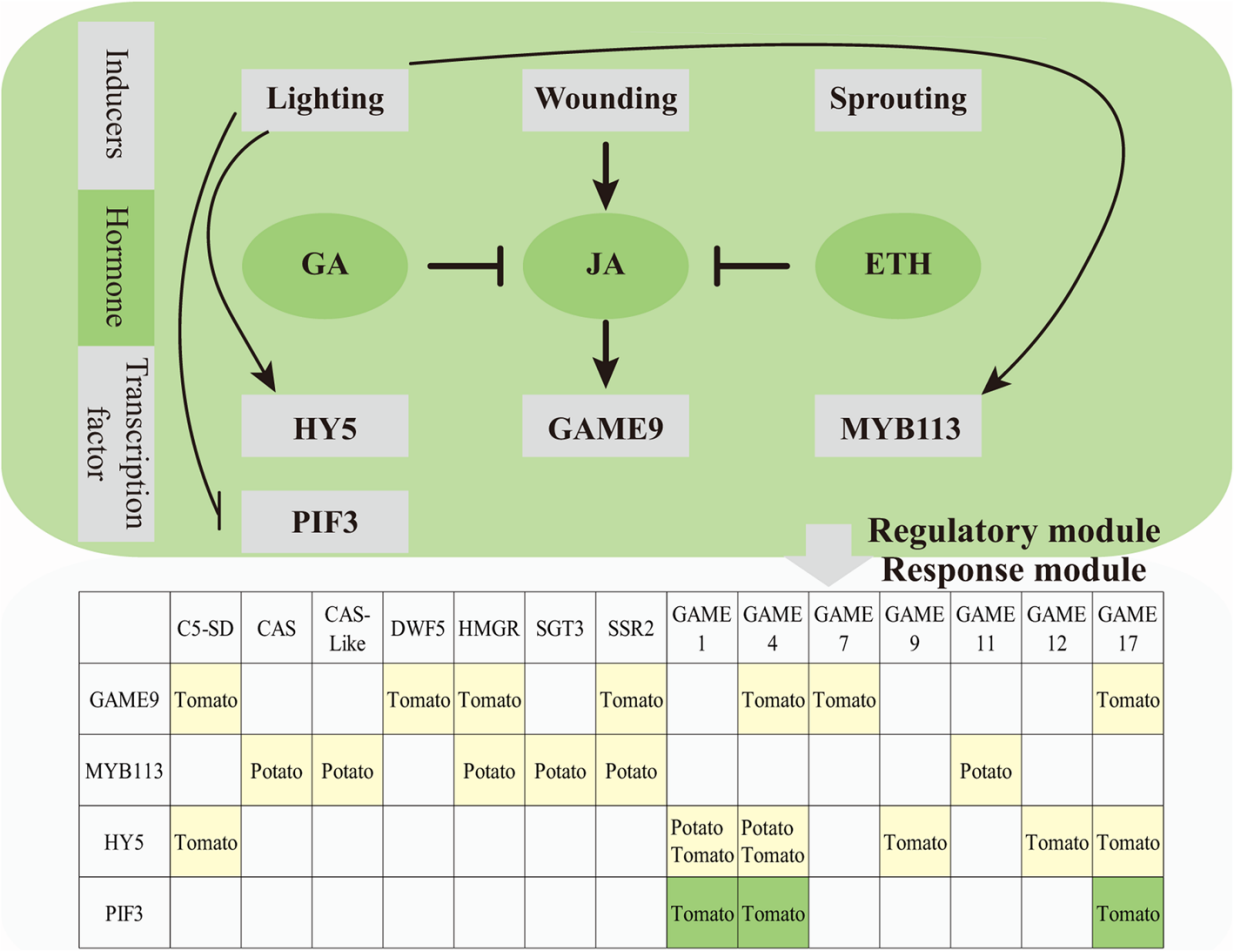
Regulatory and response modules and their crosstalk in the biosynthesis of potato steroidal glycoalkaloids (SGAs). In the regulatory module, arrows represent activation effects, and termination symbols represent inhibitory effects. In the response module, yellow represents activation, and green represents suppression. “Potato” and “Tomato” indicate the function verification chassis

TFs can regulate the expression of target genes in a sequence-specific manner by binding to their promoter regions. Currently, identified transcription factors that regulate potato glycoalkaloid synthesis include light signal transduction factors (HY5, PIF3, and StMYB113) (Wang et al. 2018) and the AP2/ERF transcription factor GAME9 (also known as JRE4) (Fig. 4) (Abdelkareem et al. 2017; Montero-Vargas et al. 2018; Swinnen et al. 2022).*GAME9* co-expresses with 37 genes involved in SGA biosynthesis and activates the transcription of downstream genes such as *DWF5*, *C5-SD*, *GAME4*, and *SSR2* by binding to elements like the GCC-box, G-box, and GC-rich regions. Additionally, in cooperation with the jasmonic acid signal transduction factor MYC2, it can enhance the expression levels of genes such as *HMGR1*, *GAME7*, and *GAME17* (Cárdenas et al. 2016; Thagun et al. 2016; Nakayasu et al. 2018; Yu et al. 2020; Swinnen et al. 2022). In plants overexpressing *StGAME9*, the expression levels of genes involved in the SGA synthesis pathway, including *CAS*, *SSR2*, *C5-SD*, *GAME11*, *GAME6*, *GAME4*, *GAME12*, *GAME1*, *GAME2*, and *SGT2*, showed significant upregulation (Cárdenas et al. 2016). Simultaneously, the content of α-solanine and α-chaconine in leaves and tuber peel increased by varying degrees, ranging from 1 to 5-fold (Cárdenas et al. 2016). These results indicate that *GAME9* is a key regulatory factor in the biosynthesis of glycoalkaloids. Through comparative transcriptome and proteome analyses of four potato varieties during the light-induced greening process, Liu et al. (Liu et al. 2024) identified StMYB113 as a positive regulator of steroidal glycoalkaloid biosynthesis. Dual luciferase assays indicated that StMYB113 could bind to and activate the promoters of genes related to steroidal glycoalkaloid biosynthesis, such as *CAS-Like*, *CAS*, *GAME11*, *HMGR*, *SGT3*, and *SSR2*. The overexpression of StMYB113 significantly increased the levels of SGAs and the expression of *StGAME11* and *StGAME9*.

Recent research has unveiled a molecular network involving the “Transcription Factor-Enhancer-Promoter” system, which regulates the metabolism of glycoalkaloids in tomato fruits. The distal enhancer, known as GAME Enhancer 1 (GE1), recruits the MYC2-GAME9 transcriptional complex, facilitating enhancer-promoter chromatin looping. This process spatially regulates the expression of the GAME gene cluster located on chromosome 7, thereby modulating the metabolism of SGAs (Bai et al. 2024). Furthermore, various transcription factors, including TAGL1 (Zhao et al. 2018), TDR4 (Zhao et al. 2019), MYB12 (Chen et al. 2019), bHLH114 (Li et al. 2020), MYC (Swinnen et al. 2022), DELLA (Panda et al. 2022), SlERF.D6 (Guo et al. 2022), SlERF.H6 (Hao et al. 2023), and SlDOG1 (Zhao et al. 2023), have been implicated in the regulation of α-tomatine biosynthesis in tomatoes. However, their regulatory roles in the biosynthesis of SGAs in potatoes require further validation.

### Regulation by hormones

#### Jasmonic Acid (JA)

JA is a critical class of lipid hormones produced by plants, playing a pivotal role in plant growth, development, and stress responses. Studies have shown that the exogenous application of methyl jasmonate (MeJA) can stimulate the increase of SGA levels in tubers (Abdelkareem et al. 2017). Injury frequently induces an increase in endogenous jasmonic acid levels, which may explain the phenomenon of elevated SGA levels triggered by damage (Petersson et al. 2013; Nahar et al. 2017; Nie et al. 2019; Shen et al. 2022; Merino et al. 2023). The mechanism by which jasmonic acid regulates SGA biosynthesis is primarily investigated in tomatoes. Studies have shown that the same members of signaling pathways are present in the synthesis pathways of SGA and JA. The disruption of the function of JA biosynthetic genes *AOC* (allene oxide cyclase), *JAR1* (JASMONATE RESISTANT 1), JA receptor component F-box protein *COI1* (CORONATINE INSENSITIVE 1), as well as signaling transduction factors *MYC1* and *MYC2*, influences the biosynthesis of SGAs (Abdelkareem et al. 2017; Panda et al. 2022). Furthermore, it has been found that the JA signaling negative regulatory factors *JAZ1*, *JAZ2*, and *JAZ6* negatively regulate the biosynthesis of SGAs (Panda et al. 2022). Currently, research has revealed that the SGA synthesis genes *C5-SD*, *GAME4*, *GAME7*, and *HMGR1* are cooperatively regulated by the jasmonic acid signaling factors MYC2 and GAME9 (JRE4) (Cárdenas et al. 2016; Swinnen et al. 2022). Moreover, other genes including *HMGR1*, *SMO2*, *DWF5-2*, and *SMO4* also show MeJA-induced expression (Thagun et al. 2016; Nakayasu et al. 2018).

#### Gibberellic Acid (GA)

GAs are diterpene plant hormones involved in various processes of plant growth and development, including the regulation of seed germination, stem elongation, and the development of flowers and fruits. In potato production, GAs are utilized to break dormancy in potato tubers and stimulate tuber sprouting. There is a positive correlation between the levels of SGAs and GAs in potato plants. Exogenous gibberellin treatments significantly boosted SGA content in various organs such as tubers, roots, stems, and leaves (Abdullah et al. 1980; Jia et al. 2019). Conversely, in tomatoes, blocking gibberellin biosynthesis promoted the accumulation of α-tomatine. The levels of α-tomatine increased in the gibberellin receptor mutant gid1^TRI^ and decreased in the negative regulatory factor DELLA protein mutant procera (pro). Furthermore, the expression levels of genes such as *SSR2*, *GAME1*, *GAME17*, *GAME11*, and *GAME12* were negatively correlated with gibberellin signaling in tomatoes (Panda et al. 2022). The contrasting responses of SGA accumulation to GA signals between potato and tomato may be attributed to their varying sensitivities to GA concentrations, highlighting further comprehensive studies. Gibberellins and jasmonic acid are antagonistic hormones that regulate plant growth and defense. Consequently, gibberellins may regulate SGA synthesis by influencing jasmonic acid signal transduction.

#### Ethylene (ETH)

ETH is a vital plant hormone that plays a crucial role in plant growth, development, and various physiological processes, including stress responses, fruit ripening, and leaf abscission. Studies have demonstrated that a small amount of ethylene promotes the accumulation of SGAs in excised tubers, while a higher amount inhibits SGA accumulation (Bergenstråhle et al. 1992). In tomatoes, ethylene facilitates the conversion of α-tomatine to esculeoside A in mature fruits (Iijima et al. 2009). Transcript level analysis indicates that ethylene inhibits the induction of SGA biosynthesis genes by JA (Nakayasu et al. 2018; Shoji et al. 2022). Further research is necessary to clarify the precise mechanism of ethylene action in SGA metabolism.

### Light regulation of the biosynthesis of SGAs in potato

Potatoes inevitably experience light exposure during post-harvest processes, including transportation, processing, and sales. Prolonged periods of light exposure can lead to a significant increase in the content of SGAs in tubers (Percival. 1999; Zrust et al. 2001; Chuda et al. 2004; Baur et al. 2022). In potatoes undergoing light-induced greening, the SGA content can rise from 0.004 to 0.08%, representing a 20-fold increase (Petersson et al. 2013). Investigation has established that different light qualities exert varying effects on SGA metabolism. In contrast to darkness and far-red light, red and blue light significantly stimulate the accumulation of α-solanine and α-chaconine in tubers (Okamoto et al. 2020), while yellow light can significantly inhibit glycoalkaloid accumulation (Mekapogu et al. 2016). Moreover, potato varieties exhibit varying sensitivities to light-induced SGA accumulation (Petersson et al. 2013; Nahar et al. 2017; Baur et al. 2022; Merino et al. 2023). Additionally, light exposure and mechanical damage have been shown to exert a synergistic effect on SGA accumulation in tubers (Nie et al. 2019).

Light-induced substantial accumulation of SGAs presents a significant challenge for maintaining the post-harvest quality of potatoes. Therefore, understanding the mechanisms of light signal regulation on SGA metabolism is very crucial. Investigation found that exposure to light treatment significantly increases the expression levels of numerous SGA synthesis genes in tubers, including *HMGR1*, *SQS*, *CAS1*, *SSR2*, *SGT1*, *SGT2*, *SGT3*, *GAME4*, *GAME6*, *GAME11*, and *GAME12* (Nahar et al. 2017; Zhang et al. 2019; Okamoto et al. 2020). ELONGATED HYPOCOTYL 5 (HY5) and PIF3 serve as positive and negative regulatory factors in photomorphogenesis, respectively. The expression of HY5 increases under visible light or UV-B conditions but decreases in darkness. Conversely, PIF3 is degraded in light, leading to increased levels in the dark. In the tomato CRISPR-mediated knockout mutant hy5, the transcript levels of genes such as *GAME1*, *GAME2*, *GAME6*, *GAME11*, *GAME18*, and *GAME25* are downregulated, resulting in reduced levels of eight glycoalkaloids, including α-tomatine (Zhang et al. 2022a). Conversely, overexpression of HY5 leads to increased (Sinha et al. 2024). Experiments revealed that HY5 could bind to the promoters of *SlGAME1/StGAME1*, *SlGAME4*/*StGAME4*, *SlGAME11*, *SlGAME12*, *SlGAME17*, *SlC5SD*, and *SlGAME9*, respectively (Wang et al. 2018; Sinha et al. 2024; Chao et al. 2024). Dual-luciferase reporter assays demonstrate that HY5 promotes the expression of *GAME1*, *GAME4*, and *GAME17*, thus playing a positive regulatory role in SGA biosynthesis. In contrast, PIF3 inhibits the expression of these genes, exerting a negative regulatory role in SGA accumulation (Wang et al. 2018). However, due to the lack of a typical transactivation domain, HY5 often relies on other transcription factors to exert its transcriptional regulatory function (Gangappa et al. 2016). Recently, SlBBX20 was reported to directly interact with HY5, and the levels of α-tomatine and dehydrotomatine were significantly reduced in its loss-of-function mutants (Shiose et al. 2024). In the future, more collaborators of HY5 in the light-regulated biosynthesis of solanine remain to be discovered.

### Non-coding RNA

Non-coding RNA (ncRNA) refers to all RNA molecules in the cell that do not encode proteins, mainly including ribosomal RNA (rRNA), transfer RNA (tRNA), small RNA, and long non-coding RNA (lncRNA). MicroRNA (miRNA) is an important post-transcriptional regulatory factor in plants, playing a crucial role in plant development by degrading target gene transcripts or interfering with their translation. Studies have found that miRNA may participate in the metabolism of SGAs in potatoes through the JA signaling pathway, uridine diphosphate glucose (UDP-glucose) biosynthesis, and hydroxylation reactions (Qiao et al. 2017, 2023). However, there is no direct evidence showing that ncRNA affects SGA metabolism, making it essential to explore this relationship for a better understanding of the regulation of SGA synthesis in future research.

## Genetic improvement of SGAs in potato

Regulating the levels of SGAs in potato tubers represents a crucial objective in breeding programs. Biotechnology offers two potential avenues to suppress SGA accumulation in plants. The first approach involves disrupting the normal function of SGA synthesis genes or regulators to block SGA production. Currently, approximately 30 genes have been identified as directly involved in SGA synthesis, however, genes whose loss would result in detrimental effects should be excluded. For instance, silencing *PGA1* or *PGA2* can lead to plant sterility and inhibit tuber sprouting (Umemoto et al. 2016), while reduced expression of *16DOX* can prolong tuber dormancy (Nakayasu et al. 2017). *GAME4* represents a suitable target gene, as silencing *GAME4* can decrease SGA content in leaves and tubers by up to 74-fold without inducing apparent growth and developmental defects (Itkin et al. 2013; Sawai et al. 2014). As a key regulatory factor, silencing *GAME9* dramatically reduces SGA content in potatoes (Cárdenas et al. 2016; Thagun et al. 2016; Nakayasu et al. 2018; Yu et al. 2020; Swinnen et al. 2022). Moreover, compared to RNA interference (RNAi) method, CRISPR technology can completely inhibit SGA synthesis. For example, while RNA silencing of *16DOX* significantly reduces SGA levels in plants (Nakayasu et al. 2017), employing the CRISPR system to knock out *16DOX* can completely suppress SGA synthesis (Nakayasu et al. 2018). The second approach involves introducing SGA degradation genes to further convert SGAs into non-bitter and non-toxic compounds. As a close relative of potatoes, unripe tomato fruits also accumulate massive SGAs (primarily α-tomatine). The hydroxylase GAME31/23DOX, glycosyltransferase GAME5, together with other unidentified enzymes can transform α-tomatine into non-toxic substances, thereby rendering ripe tomatoes devoid of bitterness and toxicity (Cárdenas et al. 2019; Nakayasu et al. 2020; Szymański et al. 2020). The knockout of tomato *7*-*dehydrocholesterol reductase 2* (*7*-*DR2*) facilitates the reduction of α-tomatine and the accumulation of provitamin D3 without evident adverse effects on the plants (Li et al. 2022). Considering the similarity in SGA biosynthesis steps and transcriptional regulation between potatoes and tomatoes (Fig. 3), the metabolic pattern of SGAs in tomato fruits may provide insights into converting potato SGAs into non-toxic substances. Furthermore, the discovery of catalytic enzymes capable of completely degrading α-solanine and α-chaconine in certain microorganisms presents a promising avenue for future research (Hennessy et al. 2020; Song et al. 2023).

## Future perspectives

### Comprehensive analysis of the functions of SGAs

Glycoalkaloids have been demonstrated to exhibit multiple functions, including inhibitory effects on diseases, pests, and tumors (Jiang et al. 2016; Cárdenas et al. 2019; Fabian et al. 2023). In addition to the primary glycoalkaloids α-solanine and α-chaconine, further exploration and utilization of the functional roles of other SGAs in potatoes using emerging techniques (Yang et al. 2024) and population analysis need to be carried out (Zhang et al. 2022b; Guo et al. 2023). Notably, a recently identified tetraose SGA in wild potatoes exhibits resistance to *Alternaria solani* and the Colorado potato beetle, while demonstrating inhibitory effects on various pathogenic fungi affecting potatoes (Wolters et al. 2023).

### In-depth study of the regulatory network of SGAs synthesis

Although most genes involved in the biosynthesis of SGAs have been identified, the transcriptional regulatory mechanisms controlling these genes are not yet fully understood. Plant metabolism is often regulated by various upstream pathways (Yan et al. 2022), and the metabolic regulation of SGAs at multiple levels remains to be elucidated. These levels include transcriptional regulation involving transcription factors and DNA methylation, post-transcriptional regulation, post-translational modifications such as phosphorylation, acetylation, ubiquitination, and glycosylation, epigenetic regulation, and other complex regulatory networks.

### Unveiling the metabolic connection between SGAs and other substances

The variation in solanine glycoalkaloid (SGA) content in potatoes can induce alterations in various primary and secondary metabolites. As glycoalkaloids accumulate in tuber buds, differential expression occurs in genes involved in plant hormone signaling, carbohydrate metabolism, and secondary metabolite biosynthesis. Consequently, levels of lipids, amino acids and their derivatives, phenolic acids, alkaloids, hormones, and other metabolites also exhibit significant changes (Shen et al. 2022; Sinha et al. 2024). Furthermore, SGA accumulation can trigger extensive metabolic changes throughout the entire plant via transcriptional reprogramming (Cárdenas et al. 2016). These observations collectively delineate a complex regulatory network.

### Toward the rational design of SGAs

Optimizing SGA metabolism is a crucial direction in potato breeding to enhance edibility, safety, and resistance to diseases and pests. Utilizing target genes cloned from wild potatoes can catalyze the conversion of triose SGAs to tetrose SGAs, thereby conferring broad-spectrum disease and pest resistance to transgenic plants (Wolters et al. 2023). The 2-ketoglutarate-dependent dioxygenase GAME32 derived from wild potatoes can hydroxylate potato α-chaconine and α-solanine into leptinines, which are non-bitter and resistant to beetles (Cárdenas et al. 2019). Similar to α-tomatine in tomatoes (Kazachkova et al. 2021) and glucosinolates in rapeseed (Xu et al. 2023), deciphering the intercellular transport mechanism of potato SGAs will help eliminate anti-nutritional factors in the edible portion. Additionally, high-productivity varieties often produce a large number of berries, and tissue-specific removal of SGAs holds the promise of developing potato berries into new food resources (Liu et al. 2023; Silva et al. 2024). Since potatoes contain more than 80 glycoalkaloids, we also need to examine the content of other glycoalkaloids after eliminating α-solanine and α-chaconine. Furthermore, along with traits such as self-incompatibility, tuberization under short day conditions, and long stolons, high SGA content is one of the key traits for the de novo domestication of wild potatoes (Egorova et al. 2022).

## Data Availability

Not applicable.

## Abbreviations

acetyl-CoA: Acetyl-coenzyme A
AOC: Allene oxide cyclase
CAS: Cycloartenol synthase
COI1: Coronatine insensitive 1
CYP: Cytochrome P450 monooxygenase
DMAPP: Dimethylallyl diphosphate
DMF: N, N-dimethylformamide
DPS: Dioxygenase for potato solanidane synthesis
DWF1-L: Δ24-Sterol reductase-like
DWF5-L: ∆7-Sterol reductase-like
DWF7-L: ∆7-Sterol C5-desaturase-like
ETH: Ethylene
FW: Fresh weight
GA: Gibberellin
GAME: Glycoalkaloid metabolism
GE1: GAME Enhancer 1
HY5: ELONGATED HYPOCOTYL 5
HMGR1: 3-Hydroxy-3-methylglutaryl-coenzyme A reductase 1
IPP: Isoprenoid pyrophosphate
JA: Jasmonic acid
JAR1: Jasmonate resistant 1
lncRNA: Long non-coding RNA
MeJA: Methyl jasmonate
miRNA: MicroRNA
MVA: Mevalonate
ncRNA: Non-coding RNA
RNAi: RNA interference
rRNA: Ribosomal RNA
SGA: Steroidal glycoalkaloid
SGT: Solanidine galactosyltransferase
SMO1-L: Sterol C4-methyl oxidase 1-like
SQE: Squalene epoxidase
SQS: Squalene synthase
SSR2: Sterol side chain reductase 2
TF: Transcription factor
TGA: Total glycoalkaloid
tRNA: Transfer RNA
UDP-glucose: Uridine diphosphate glucose
UGT: UDP-glycosyltransferase
7-DR2: 7-Dehydrocholesterol reductase 2
